# Shelf-Life Extension of Large Yellow Croaker (*Larimichthys crocea*) Using Active Coatings Containing Lemon Verbena (*Lippa citriodora* Kunth.) Essential Oil

**DOI:** 10.3389/fnut.2021.678643

**Published:** 2021-07-20

**Authors:** Bo Li, Xuesong Wang, Xin Gao, Xuan Ma, Leilei Zhang, Jun Mei, Jing Xie

**Affiliations:** ^1^College of Food Science and Technology, Shanghai Ocean University, Shanghai, China; ^2^National Experimental Teaching Demonstration Center for Food Science and Engineering Shanghai Ocean University, Shanghai, China; ^3^Shanghai Engineering Research Center of Aquatic Product Processing and Preservation, Shanghai, China; ^4^Shanghai Professional Technology Service Platform on Cold Chain Equipment Performance and Energy Saving Evaluation, Shanghai, China; ^5^School of Health and Social Care, Shanghai Urban Construction Vocational College, Shanghai, China; ^6^Shanghai Guo Qi Testing Services Technology Co., Ltd., Shanghai, China

**Keywords:** active coating, essential oil, large yellow croaker, total volatile basic nitrogen, shelf-life extension

## Abstract

Active coating could improve the fish quality and extend the shelf life. This study investigates the effect of locust bean gum (LBG) and sodium alginate (SA) active coatings containing lemon verbena (*Lippa citriodora* Kunth.) essential oil (LVEO) emulsions on microbiological, physicochemical and organoleptic evaluation of large yellow croaker (*Larimichthys crocea*) samples during refrigerated storage at 4°C. Results showed that LBG-SA coatings incorporated with 0.30 or 0.60% LVEO emulsions significantly inhibited the growth of mesophile bacteria, *Pseudomonas* spp., H_2_S-producing bacteria, lactic acid bacteria (LAB) and psychrophilic bacteria, and reduce the productions of trimethylamine (TMA), total volatile basic nitrogen (TVB-N) and ATP-related compounds. Further, the LVEO treatments also retarded the water migration and maintained the organoleptic evaluation results of large yellow croaker during storage at 4°C. In conclusion, the LBG-SA active coatings incorporated with LVEO emulsions maintained the quality and extended the shelf life of large yellow croaker during refrigerated storage.

## Introduction

Large yellow croaker (*Larimichthys crocea*) is an important commercial marine fish in China and cultured extensively due to its flavor and commercial value (1, 2). However, fresh large yellow croaker is highly perishable and results in great economic losses (3, 4) due to lipid oxidation, protein degradation, and the production of undesirable compounds in the presence of microorganisms and related enzymes (5). Freshness is the most important issue relating to its quality and value (6). The fish spoilage could produce trimethylamines (TMA), organic acids, biogenic amines, alcohols, sulfides, ketones and aldehydes with unacceptable off-flavors. Fish spoilage is mainly related to the presence of Gram-negative proteolytic psychrotrophic bacteria, mainly *Pseudomonas* spp., *Shewanella* spp., and *Enterobacteriaceae* (7).

Some preservation techniques have applied to improve the quality and extend the shelf life (8). Using active coatings to delay the microbial growth on the fish surface could improve the fish quality and extend its shelf-life (9). Active coating mainly from food-grade natural materials including polysaccharides (e.g., alginates, gum, chitosan) has been developed as the coating for fish and fish products (10). Natural plant preservative has been paid more and more attention as it have little impact on human health or the environment to control spoilage organisms (11). Sodium alginate (SA) is an anionic polysaccharide and has good film forming properties (12). However, pure SA film still has relatively poor mechanical strength and antimicrobial activity, limiting its application to food packaging if modified properly (13). Locust bean gum (LBG) is a natural high molecular weight (300–1,200 kDa) branched polysaccharide (14). Being non-ionic, its aqueous solubility is not affected by pH or ionic strength of the liquid medium (15). SA could be used as an ion source (anionic) to promote the mucoadhesive property of non-ionic LBG (15, 16).

The incorporation of natural bioactive compounds in the active coating could improve the quality and performance, and essential oils (EOs) are the commonly used categories. Lemon verbena (*Lippa citriodora* Kunth.) is an aromatic plant native to South America and widely used for medicinal purposes, including antimicrobial, neuroprotective, anticonvulsant, cardioprotective, antigenotoxic, and anti-inflammatory activities (17, 18). Previous research has shown that high percentage of neral in LVEO exhibits antimicrobial and antioxidant activities (19). Nevertheless, the antimicrobial activity of neral against several spoilage organisms has been well-documented in *in-vitro* trials and applied in food storage (20, 21). However, some chemically active compounds of EOs are rarely present in food matrices, which have negative impact on the chemical food integrity, physical stableness, and the loss of bioactive activity of bioactive compounds (22). LVEO encapsulated by emulsion can conquer these problems by improving the oxidative stability of compounds, limiting the reaction of these compounds with food, protecting their constancy during process of food and maintenance, and providing controlled and targeted release conditions (22–24). Biopolymeric emulsions with high food compatibility could inhibit the microbial growth and lipid oxidation in fish during cold storage.

The research was to explore the effect of LBG and SA based coatings incorporated with LVEO on the quality of refrigerated large yellow croaker. The changes in microbial survival, total volatile basic nitrogen (TVB-N), trimethylamine (TMA), *K*-value, lipid oxidation, free fatty acids, hardness, and organoleptic evaluation of refrigerated large yellow croaker during storage for 18 days were tested to determine the preservative mechanism of each treatment.

## Materials and Methods

### Essential Oil From Lemon Verbena

The leaves of lemon verbena were washed with deionized water and then hydro-distillated by a Clevenger-type apparatus for 3 h. LVEO was dried with sodium sulfate anhydrous and then kept at 4°C in sealed brown vials till being used. The LVEO components were analyzed by GC-MS with the method of Homayonpour et al. (10). The conditions were set as follow: Sample volume: 1 μL; Injection port temperature: 280°C; Ion source temperature: 230°C; Initial temperature: 60°C for 1 min; Program rate: 10°C/min; Final temperature: 290°C for 5 min; Septum purge with flow rate 2 mL/min. The LVEO components were recognized with confirmed with those of mass spectra and authentic samples with reference compounds in the NIST 2011. The relative content of each component of LVEO (%) was measured with area under peak.

### Preparation of Active Films Incorporated With LVEO Emulsions

The LVEO/lecithin emulsions were prepared according to Liu et al. (16). The minimum inhibitory concentration (MIC) and minimum bactericidal concentration (MBC) of LVEO for *Pseudomonas* spp. and *Shewanella* spp. are 0.30 and 0.60%, respectively. Therefore, different concentrations of LVEO (0.15, 0.30, and 0.60%, v/v considering the 1/2 MIC and MIC, as well the MBC concentrations, respectively) and 0.15% (w/v) lecithin were stirred mechanically in a beaker. Then the LVEO/lecithin emulsions were homogenized with a rotor-stator homogenizer (HR-6, Huxi Industrial co., LTD, Shanghai, China) at 15,000 rpm for 5 min. LBG-SA solution was prepared with SA (1.5% w/v, *M*/*G* = 2:1, Mw 2.1 × 10^6^ g/mol, viscosity of 200 ± 20 mPa•s, specifications received from supplier) and LBG (0.5% w/v, from *Ceratonia siliqua* seeds, >75% galactomannan content, *M*/*G* = 4:1, Mw 320 kDa, specifications received from supplier). To obtain complete dispersion of LBG-SA, the solution was stirred at 60°C for 4 h. Glycerol (30% w/w based on LBG-SA) as a plasticizer was added to LBG-SA solution and stirred for 1 h. The pH value of 1.5% w/v sodium alginate and 0.5% w/v locust bean gum solutions were about 6.7 and 6.2, respectively. The resultant LBG/-SA coating solution was filtrated through a double-layer degreased gauze to remove any undissolved particles. The prepared LVEO/lecithin emulsions were separately added to the LBG-SA solution and stirred continuously for 2 h. Then, the LBG-SA active coatings solutions incorporated with LVEO emulsions were prepared with ultrasonic assisted treatments at 700 W using a ultrasonic assisted processor and degassed under vacuum.

### Preparation of Large Yellow Croaker Samples

Fresh large yellow croaker (700 ± 25 g) were supplied by a local market and randomly divided into four batches. Each batch of samples was immersed in the corresponding freshly prepared active coating solutions for 20 min with a ratio of 1:3 (w/v). Then the large yellow croaker samples were taken out and air-dried at 4°C for 60 min to form the active coating. After that, each large yellow croaker sample was packaged in sterile polyethylene bag and stored at 4°C for the subsequent assessments at 3-day interval. The abbreviation was followed: (1) CK (large yellow croaker samples were treated with LBG-SA active coating without LVEO emulsion); (2) LYC-0.15%LVEO (large yellow croaker samples were treated with LBG-SA active coating incorporated with 0.15% LVEO emulsion); (3) LYC-0.30%LVEO (large yellow croaker samples were treated with LBG-SA active coating incorporated with 0.30% LVEO emulsion); (4) LYC-0.60%LVEO (large yellow croaker samples were treated with LBG/-SA active coating incorporated with 0.60% LVEO emulsion).

### Microbiological Analysis

Twenty-five grams fish flesh were fully blended with 225 mL normal saline and then subjected to gradient dilutions. The following microbiological analyses were carried out (25): (i) mesophile bacteria: plate count agar mediums were cultivated at 30°C for 48 h; (ii) *Pseudomonas* spp.: cetrimide agar mediums were cultivated at 30°C for 72 h; (iii) H_2_S-producing bacteria: iron agar mediums were cultivated at 30°C for 72 h; lactic acid bacteria: MRS agars were cultivated at 30°C for 72 h; (v) psychrophilic bacteria: plate count agar mediums were cultivated at 30°C for 7 days. Each sample was measured in triplicates.

### Total volatile Basic Nitrogen Determination

TVB-N determination was carried out with the method of Zhuang et al. (26). 5.0 g of minced fish flesh and 45 mL deionized water were homogenized and then centrifuged at 3,040 × g at 4°C for 5 min. 5.0 mL of the supernatant was taken to determine the content of TVB-N using steam distillation method with Kjeldahl equipment (Kjeltec 8400, Foss, Denmark) and TVB-N expressed as mg N/100 g of large yellow croaker muscle. Each sample was measured in triplicates.

### Determination of Trimethylamine

TMA value was determined by picric acid colorimetric method according to Li et al. (27). 2.0 g of minced fish flesh and 18 mL trichloroacetic acid (7.5%, w/v) were homogenized and then centrifuged at 11,960 × g at 4°C for 10 min. After that, 5.0 mL of the supernatant were mixed successively with 1 mL of formaldehyde (10%, v/v), 10 mL of anhydrous toluene and 3 mL of saturated potassium carbonate solutions. Subsequently, 5 mL solution extracted from toluene layer was fully blended with 5 mL of picric acid solution (0.02%, w/v). The absorbance of the mixture was measured at 410 nm. TMA content was calculated according to TMA standard curve and expressed as mg 100 g^−1^ sample. Each sample was measured in triplicates.

### Determination of *K-*Value

ATP-related compounds were determined by a RP-HPLC procedure with the method of Yu et al. (28). 2.0 g of minced fish flesh and 7.5 mL precooled perchloric acid solution (6%, v/v) were homogenized and then centrifuged at 11,960 × g at 4°C for 5 min. The precipitate was extracted again with the same condition. The supernatants were collected and neutralized with KOH solutions to the final pH ranges of 6.5–6.8. After that, the neutralized solution was centrifuged at 3,040 × g at 4°C for 5 min and the supernatant was made up to 25 mL with deionized water. Subsequently, the prepared solution was filtered through a 0.22-μm filter membrane and analysized using HPLC (Waters 2695, Milford, USA) furnished with a Shim-pack VP-ODS C18 column (150 × 46 mm). Each sample was measured in triplicates. *K*-value was calculated according to Equation (1).

(1) $$\begin{array}{l} K\;value\;(\%)=\frac{HxR+Hx}{ATP+ADP+AMP+IMP+HxR+Hx}\\ \;\times 100 \end{array}$$

where HxR, Hx, ATP, ADP, AMP, IMP, are hypoxanthine riboside, and hypoxanthine, adenosine triphosphate, adenosine diphosphate, adenosine monophosphate, inosine monophosphate, respectively.

### Determination of Peroxide Value

POV was determined by the method described by Quan et al. (29). 1.0 g of minced fish flesh and 11 mL of chloroform/methanol (2:1, v/v) were homogenized and then centrifuged at 11,960 × g at 4°C for 2 min. 7.0 mL of the supernatant and 2 mL of 0.5% sodium chloride solution were homogeneously mixed and then centrifuged at 3,040 × g at 4°C for 5 min to separate the solution into two phases. Three milliliter of lower phase, 2 mL of chloroform/methanol (2:1, v/v), 25 μL of ammonium thiocyanate and 25 μL of iron (II) chloride were mixed uniformly. The reaction mixture stood for 20 min at room temperature for 20 min and the absorbance was measured at 500 nm. Each sample was measured in triplicates. Results were expressed in mmol mequiv. peroxide/100 g sample:

(2) $$POV=\frac{V\times N\times 1000}{W}$$

V is the thiosulphate for titration (mL); N is the normality of thiosulphate; W is the weight of lipid (g).

### Evaluation of Thiobarbituric Acid Reactive Substances

TBARS was monitored with the method of Vale et al. (30) and expressed as mg of malonaldehyde (MDA)/kg of large yellow croaker sample. Five grams flesh and 20 mL of 20% TBA solution were homogeneously mixed and stood for 1 h. Then the mixture was centrifugated at 11,960 × g at 4°C for 10 min and collected the supernatants. Five milliliter collected supernatant was mixed with 5 mL TBA (0.02 M) and boiled for 40 min. Then the mixture was immediately transferred to ice bath and the absorbance was measured at 532 nm. Each sample was measured in triplicates.

### Determination of Free Fatty Acids

The FFAs concentration of the large yellow croaker muscle lipid extract was measured by colorimetric reaction with cupric acetate-pyridine and the absorbance was determined at 715 nm according to Trigo et al. (31). The results were expressed as mmol·kg^−1^ muscle. Each sample was measured in triplicates.

### Water Distribution and Migration

Low field nuclear magnetic resonance (LF-NMR) analysis was carried out according to Li et al. (32). Portions of 2 × 2 × 1.5 cm dorsal muscle was cut off and packaged with polyethylene film. Transverse relaxation (T2) was determined on the LF-NMR analyzer (MesoMR23-060H.I, Newmai co., Ltd., China) with the proton resonance frequency of 20 MHz. The Carr-Purcell-Meiboom-Gill (CPMG) was used to obtain T_2_ relaxation information to collect decay signals. The primary parameters were as following: SW (the receiver bandwidth frequency) = 100 kHz, RFD (the parameter to control the first data point that acquired) = 0.08, NS (the number of the scans) = 4, P1 (RF 90° pulse width) = 18 μs, P2 (RF 180° pulse width) = 36 μs, RG1 (analog gain) = 20 db, DRG1 (digital gain) = 6 db, PRG (preamplifier gain) = 0, delay DL1 = 0.2 ms and TW (the duration between successive scans) = 2,000 ms. Longitudinal relaxation (T_1_) was measured by using the inversion-recovery (IR) sequence to confirm the MRI parameters followed the below parameters: P1 = 18 μs, P2 = 36 μs, SW = 200 KHz, RFD = 0.020 ms, RG1 = 20 db, DRG1 = 1, NS = 4, TW = 5,000 ms, PRG = 0, NTI = 20, and DL1 = 0.2 ms. Each measurement was performed in triplicate. Post-processing of NMR T_2_ data distributed exponential fitting of CPMG decay curves were performed by Multi-Exp Inv Analysis software (Newmai Co., Ltd., China). From the multi-exponential fitting analysis, time constants for each process were calculated from the peak position, and the area under each peak (corresponding to the proportion of water molecules exhibiting that relaxation time) was determined by cumulative integration.

After the measurement of LF-NMR, ^1^H MRI images of large yellow croaker samples were also determined on MesoMR23-060H.I NMR Analyzer (33). The MRI images, including T_1_, T_2_, and proton density weighted images, were acquired by using the spin-echo (SE) sequence. The MRI measurement was performed with time repetition (TR), slice width, and time echo (TE) being 500 ms, 3.0 mm, and 20 ms, respectively. Each sample was measured in triplicates. The MRI images were processed with two software: unified mapping and pseudocolor processing. After unified mapping and pseudocolor processing, the gray level images were converted to the color images.

### Determination of Hardness

The hardness of large yellow croaker samples was determined using a TA.XT texture analyzer equipped with P/5 probe (34). Portions of 3 × 2 × 1.5 cm dorsal muscle (about 5.0 g) was cut off and the hardness was measured with the test speed of 1 m/s and sample deformation of 50%. Each sample was measured at least eight points.

### Organoleptic Evaluation

The organoleptic evaluation of large yellow croaker samples was determined with the quality index method (QIM) described by Sun et al. (35). This method involves five important quality parameters, namely color, elasticty, mucus, muscular tissue and smell. Fifteen experienced panelists (trained by professional laboratory staff, eight women and seven men, 20–40 years old) conducted the organoleptic evaluation based on QIM with the score scale ranging from 1 to 10. All panelists were trained by professional laboratory staff and had a history in fish assessment and previously at Shanghai Ocean University, joined in another research displayed by Li et al. (36). They were designated based on their taste detection limit, sensitivity and smell of very low LVEO concentrations. Ten represents the best quality of large yellow croaker and the sample will not be accepted once the score is <4.

### Statistical Analysis

Data analysis of the quality of large yellow croaker was performed in triplicate (except hardness determination and organoleptic evaluation). The date was analyzed using SPSS 22.0 through one-way ANOVA procedure followed by Duncan's-test. The results were expressed as means ± standard deviation. *p* < 0.05 was considered to be statistically significant.

## Results and Discussions

### Chemical Composition of LVEO

The important components of LVEO used included citral (31.79%), neral (23.75%), geraniol (22.01%), and D-limonene (10.36%) obtained from the MS libraries (Supplementary Table 1). The four most abundant components are responsible of the antimicrobial activity of LVEO (37, 38).

### Microbiological Analyses

**Figure 1:**
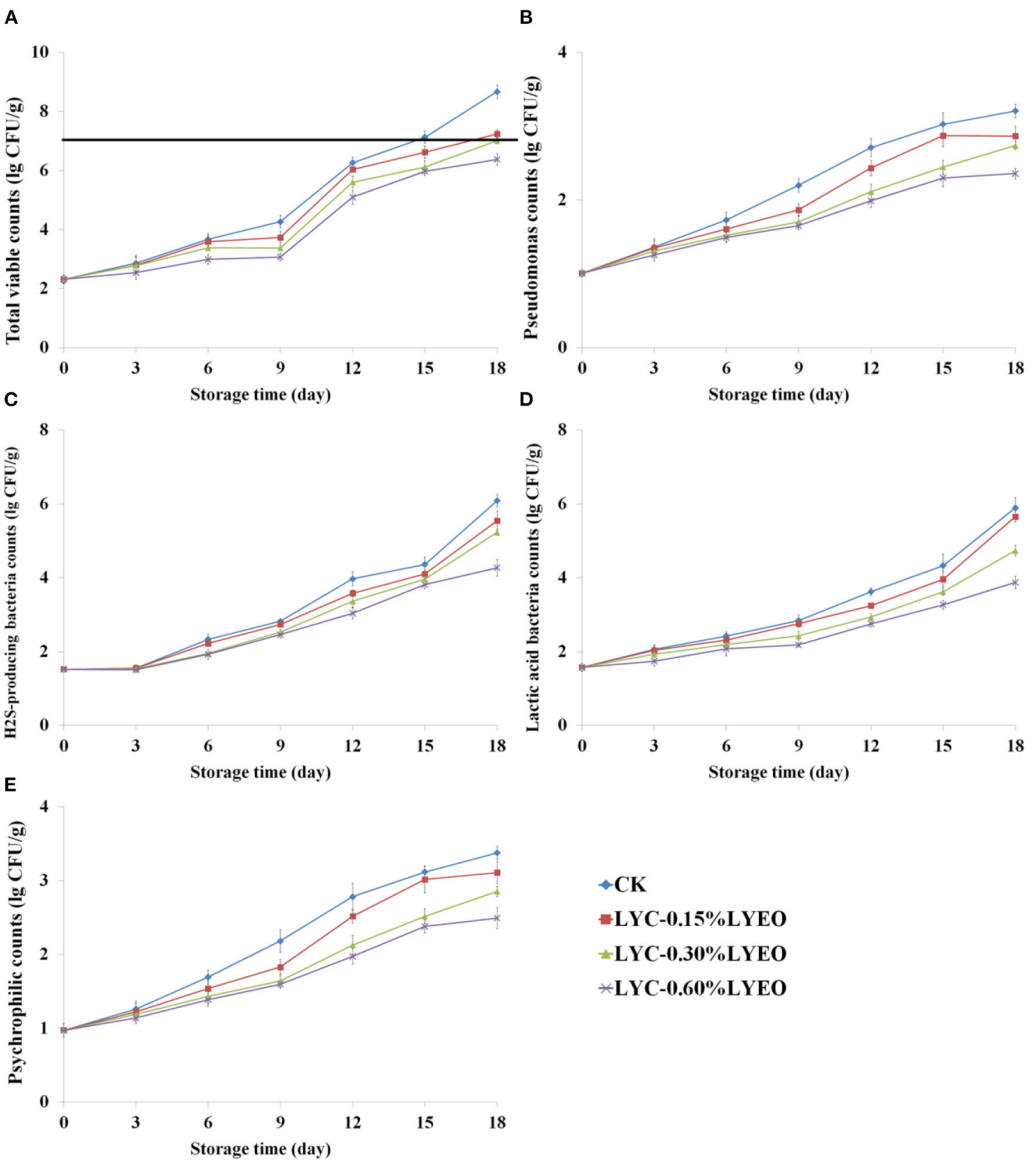
Changes in total viable counts (TVC, **A**), *Pseudomonas* spp. counts **(B)**, H_2_S-producing bacteria counts **(C)**, lactic acid bacteria counts (LAB, **D**), and psychrophilic counts **(E)** of large yellow croaker samples during refrigerated storage (CK, large yellow croaker samples were treated with LBG-SA active coating without LVEO emulsion; LYC-0.15%LVEO, large yellow croaker samples were treated with LBG-SA active coating incorporated with 0.15% LVEO emulsion; LYC-0.30%LVEO, large yellow croaker samples were treated with LBG-SA active coating incorporated with 0.30% LVEO emulsion; and LYC-0.60%LVEO, large yellow croaker samples were treated with LBG-SA active coating incorporated with 0.60% LVEO emulsion).

Figure 1 shows the corresponding growth data of TVC, *Pseudomonas* spp., H_2_S-producing bacteria, LAB and psychrophilic bacteria of large yellow croaker samples during refrigerated storage at 4°C for 18 days. The low count (2.3 lg CFU/g) of TVC at the beginning indicated fish fresh (39). LVEO treatments could delay the spoilage microbial growth and the LVEO treated large yellow croaker samples had lower TVC than that of CK. On 15th day, CK exceeded the “shelf-life” limit of 7.0 lg CFU/g (40). Incorporation of LVEO to LBG-SA coatings showed the significant antimicrobial activity of coatings, which led to extend the shelf-life by inhibiting the growth of undesirable microorganisms. EOs have been widely used for the food preservation application as having potential antimicrobial activity in active coatings (41–43). The destructions of cell membrane structure and functional characteristics of the cell membrane are considered to be the most important mechanism of EOs against microorganisms (44, 45).

*Pseudomonas* spp. and H_2_S-producing bacteria are both known as the specific spoilage organisms (SSOs) in spoiled fish during refrigerated storage (46). The two SSOs also showed similar increase results in this study (Figures 1B,C). Pseudomonas is the predominant aerobic microorganism related to the formation of undesirable odors (47). At the beginning, the number of *Pseudomonas* spp. was 1.0 lg CFU/g and increased in all large yellow croaker samples during refrigerated storage. There was a significant (*p* < 0.05) difference in the *Pseudomonas* spp. count between CK and LVEO treated samples at the end of storage and it was 8.7 lg CFU/g for CK. Therefore, it can be seen that LVEO were effective in delaying the growth of *Pseudomonas* spp. in large yellow croaker samples, and the inhibitory differences was related to the added concentration of LVEO. Nisar et al. (48) also reported that clove essential oil was also effective against *Pseudomonas* spp. and the inhibiting effects were increased with the increasing concentration of EO. Myszka et al. (49) reported that green pepper EO could inhibit the growth of *Pseudomonas* spp. and attenuate the bacterial virulence properties, such as pyocyanin production, elastase and alkaline protease activities.

The number of H_2_S-producing bacteria (mainly *Shewanella* spp.) was 1.5 lg CFU/g at the beginning, which increased to 6.1, 5.5, 5.2, and 4.3 lg CFU/g, respectively, in CK, LYC-0.15%LVEO, LYC-0.30%LVEO, LYC-0.60%LVEO, at the end of storage. The number of H_2_S-producing bacteria in LVEO treated samples were significantly (*p* < 0.05) lower than that in the CK samples. LYC-0.30%LVEO and LYC-0.60%LVEO samples had the lowest number of *Pseudomonas* spp. and H_2_S-producing bacteria in all sampling times, which indicated LBG/SA coating incorporated with 0.30 or 0.60% LVEO could inhibit the growth of the two SSOs in large yellow croaker samples during refrigerated storage. The minimum inhibitory concentration (MIC) and minimum bactericidal concentration (MBC) of LVEO for *Pseudomonas* spp. and H_2_S-producing bacteria are 0.30 and 0.60%, respectively. This is the reason that 0.30 and 0.60% of LVEO were used in the experiment. Zhang et al. (50) reported that cinnamon essential oil could also inhibit the growth of *Pseudomonas* and H_2_S-producing bacteria in in vacuum-packaged common carp during refrigerated storage and the two microorganisms did not exceed the “shelf-life” limit of 7 lg CFU/g at the end of storage. H_2_S-producing bacteria could produce fish off-odors even at low cell numbers and the spoilage action includes production of hydrogen sulfide, TMA, methyl mercaptan, and other characteristic compounds (51–53). It is reported that H_2_S-producing bacteria was responsible for putrescine and cadaverine, however, Pseudomonas spp. contributed most to tyramine (54, 55). In our previous study, H_2_S-producing bacteria grows from 2.1 to 8.9 lg CFU/g in cultured pufferfish after 18 days at 4°C, producing high levels of hexanal, 1-octen-3-ol, octanal, (E)-2-octenal and 2, 3-butanedione, which are volatile compounds causing strong fishy flavor (27).

The number of LAB was 1.6 lg CFU/g at the beginning (Figure 1D) and progressively increased in all samples during refrigerated storage but with slower rates in LYC-LVEO treated samples. The LAB number of CK, LYC-0.15%LVEO, LYC-0.30%LVEO, LYC-0.60%LVEO reached to 5.9, 5.7, 4.7, and 3.9 lg CFU/g, respectively, at the end of storage. This finding indicated that LAB was not primarily responsible for the spoilage in large yellow croaker samples during refrigerated storage, which is in consistence with trout filets packaged with probiotic carboxymethyl cellulose-sodium caseinate films (56). Although LAB was not the dominant microorganism of fish during refrigerated storage, they might cause the spoilage through producing a sour flavor and biogenic amines (47, 57).

Psychrophilic bacteria are the main microorganisms causing the spoilage of fish during refrigerated storage, thus decreasing the shelf life of fish (58). The number of psychrophilic bacteria was 1.0 lg CFU/g on 0 day and gradually increased with storage time (Figure 1E). The addition of LVEO significantly inhibited the growth of psychrophilic bacteria compared to CK (*p* < 0.05). Similar trend was found by Shokri et al. (59) with pectin based coatings containing clove essential oil.

In the current research, the counts of TVC, *Pseudomonas* spp., H_2_S-producing bacteria, LAB and psychrophilic bacteria of large yellow croaker samples packaged with LVEO active coatings were significantly lower than that of CK during refrigerated storage (*p* < 0.05). Therefore, using LVEO treatments as an active coating could maintain the microbial quality of large yellow croaker samples during refrigerated storage.

### Changes in TVB-N

**Figure 2:**
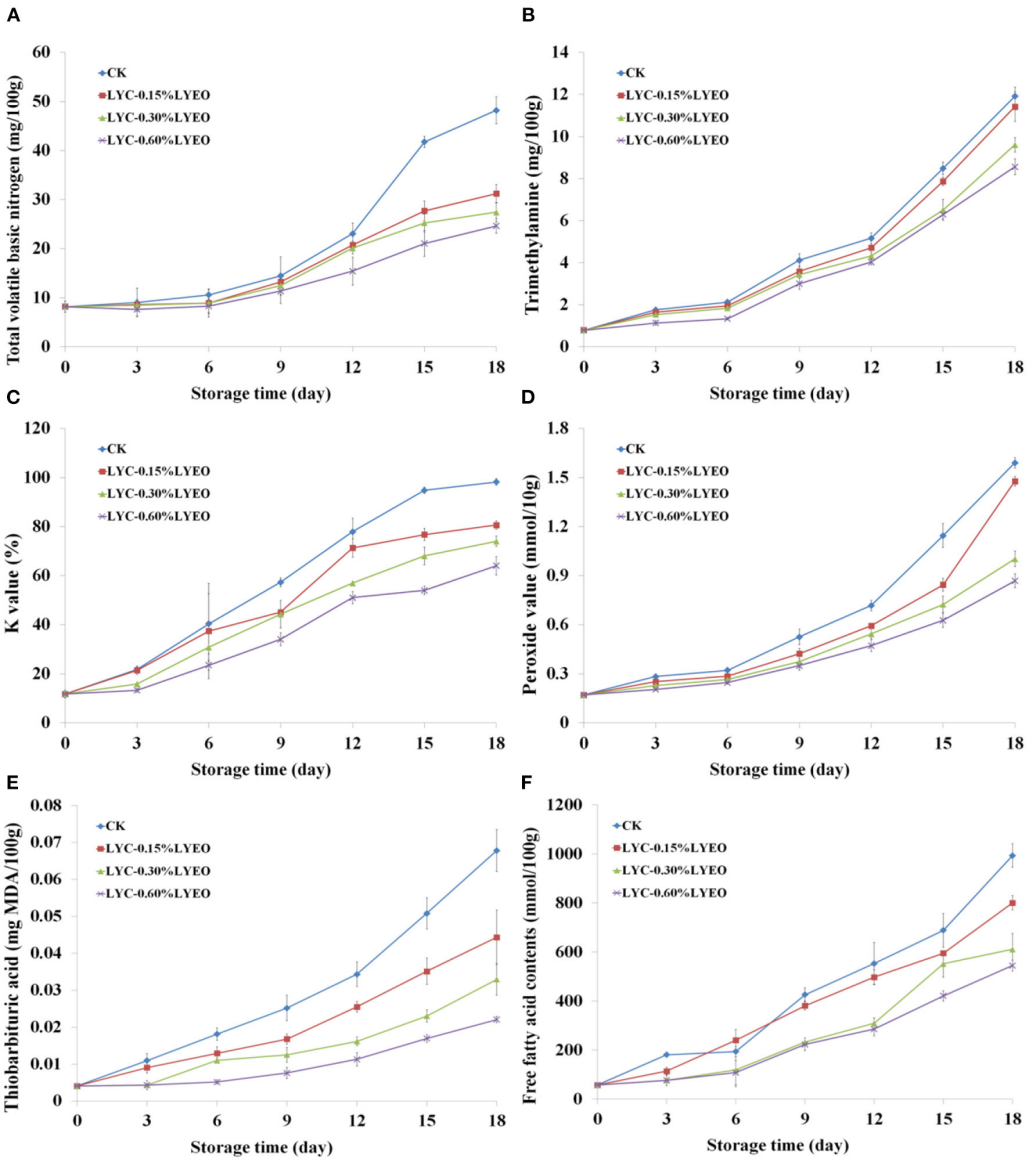
Changes in total volatile basic nitrogen (TVB-N, **A**), trimethylamine (TMA, **B**), *K*-values **(C)**, peroxide value (POV, **D**), thiobarbituric acid reactive substance (TBARS, **E**), and free fatty acids contents (FFA, **F**) of large yellow croaker samples during refrigerated storage.

Volatile nitrogen-containing compounds including ammonia, dimethylamine and TMA, known as TVB-N index, has been regarded as an indicator of spoilage in fish and fish products (60). The initial TVB-N value was determined as 8.17 mg/100 g (Figure 2A), indicting the raw fish being fresh. TVB-N contents of large yellow croaker samples progressively increased to 41.77, 27.72, and 25.26 mg N/100 g in the CK, LYC-0.15%LVEO and LYC-0.30%LVEO samples on 15th day exceeding the upper limit of 25 mg N/100 g (61). However, the LYC-0.60%LVEO samples were still under this limit at the end of storage as the 0.60% LVEO addition could inhibit the growth of SSOs or decrease the capacity of SSOs for oxidative deamination of non-protein nitrogen compounds (62). TVB-N results are consistent with TVC results. The effects of active coatings on TVB-N reduction of large yellow croaker have also been investigated. Shokri et al. (63) found that rainbow trout filets treated by chitosan with *Ferulago angulata* essential oil nanoemulsion could suppress the TVB-N growth and maintained acceptable freshness during a 16-day storage at 4°C. Dong et al. (64) also stated that active films containing Attapulgite loaded with Allium sativum essence oil pronounced lower TVB-N values in the preservation of large yellow croaker at 4°C under vacuum condition and extended shelf-life up to 9 days with 30 mg N/100 g fish as the TVB-N upper limit.

### Changes in TMA

TMA is produced through the breakdown of trimethylamine oxide (TMAO) by bacterial and enzymatic activity; therefore, it can be used as an indicator of freshness for fish and fish products (65). Low initial TMA content (0.79 mg of TMA/100 g fish muscle, Figure 2B) indicates that the large yellow croaker samples were fresh, which was consistent with the relatively low TVC counts. Moreover, H_2_S-producing bacteria could reduce TMAO to TMA and have low counts (1.5 lg CFU/g) at the beginning. The TMA of all the samples increased significantly (*p* < 0.05) during refrigerated storage. Jouki et al. suggested 5 mg N/100 g as an upper limit for rainbow trout (66). However, Klnc et al. (67) used 8 mg N/100 g as the limit of acceptability for sea bass. In the current research, the upper limit of TMA, as estimated by the TVB-N and TVC values, was 8 mg TMA/100 g for large yellow croaker samples. On the basis of this limit, CK, LYC-0.15%LVEO, LYC-0.30%LVEO, and LYC-0.60%LVEO samples exceeded the upper limit on 15th, 18th, 18th, and 18th day, respectively.

### Changes in *K-*Values

*K*-value is widely used to quantify the fish freshness. The adenine nucleotides in degradation products promote spoilage and the formation of off-flavors, causing fish to lose their freshness (68). The *K*-value of large yellow croaker samples on 0 day was 11.73% (Figure 2C), staying at a very fresh level (*K*-value <20%), and increased continuously during the whole storage time. The fish samples were considered very fresh till approximately on 6th day for the LYC-0.30%LVEO and LYC-0.60%LVEO samples, comparing with CK on 3th day, indicating that LVEO could inhibit the ATP degradation. The *K*-value of CK increased with greater speed compared with those of the LVEO treated samples. The CK, LYC-0.15%LVEO, LYC-0.30%LVEO and LYC-0.60%LVEO samples increased to 77.93, 71.33, 68.06, and 64.03% on 12th, 12th, 15th, and 18th day, respectively, exceeding the acceptable limit of 60% (69). The result was similar to Dong et al. (64), who corroborated that *Allium sativum* essence oil could effectively inhibit the ATP degradation and maintain the high quality of large yellow croaker.

### Changes in POV

POV is used to determine the formation of primary lipid oxidation products in fish and fish products during refrigerated storage (70). The POV on 0 day was 0.17 meq peroxide/kg fish (Figure 2D) and increased during refrigerated storage. The POV of all treated large yellow croaker samples increased during refrigerated storage, but at a slower rate in LVEO treated samples comparing with CK. The POV of the CK samples remarkably increased (*p* < 0.05) to 1.59 meq peroxide/kg fish at the end. This shows the CK sample was oxidized rapidly during refrigerated storage, while lipid oxidation in the LVEO treated large yellow croaker samples occurred more slowly. The delayed lipid oxidation was attributed to the release and diffusion of phenolic compounds presenting in the LBG-SA active coatings to the large yellow croaker samples. The phenolic compounds exhibited antioxidant activities, which are related with their free-radical scavenging ability and metal chelating capacities (71). These findings are consistent with those reported by Shadman et al. (72) who reported that *Zataria multiflora* Boiss. essential oil was effective in delaying the production of peroxide in rainbow trout (*Oncorhynchus mykiss*) filets during storage at refrigerated condition.

### Changes in TBARS

Fish and other seafood are rich in unsaturated fatty acids, which are easily oxidized by heat, light and enzymes, resulting in undesirable rancid odor and poisoning (73). The increase in TBA may be described by the formation of secondary lipid oxidation products (74). As shown in Figure 2E, the initial amount of MDA in the large yellow croakers was 0.04 mg MDA/kg. TBA value increased in all samples until the end of storage; however, LVEO treated samples reached significantly (*p* < 0.05) lower TBA values of 0.22–0.44 mg MDA/kg of fish comparing with CK, which attained a higher level of 0.67 mg MDA/kg of fish. A TBA level of 5 mg MDA/kg of fish muscle comprises the threshold for detecting off-odors and off-taste at refrigerated storage (56). In this research, TBA values in all samples were lower than such recommended limits during the entire storage period, which probably LBG-SA active coating can reduce the diffusion of oxygen to the surface of the fish and act as a barrier between the fish and its surroundings, thus inhibiting lipid oxidation (13). It was shown that LVEO treatments had antioxidant activity, due to a high content of neral (75), as evidenced by lower TBA values in the large yellow croaker samples packaged with LVEO coatings. Perumalla and Hettiarachchy (76) reported that the antioxidant activities of the EOs are mainly manifested in the binding of transition metal ion catalysts, the prevention of radical chain initiation, interaction with the free radicals and decomposition of peroxides.

### Changes in FFAs

Lipid hydrolysis development was measured by the FFAs formation (31). A progressive FFA formation was observed in all large yellow croaker samples during refrigerated storage (Figure 2F). However, no significant changes in FFAs contents were observed in LYC-0.30%LVEO and LYC-0.60%LVEO samples within the first 3 days. Hydrolysis of glycerol-fatty acid esters is an important change in the lipid content of muscle after fish death resulting in the release of free fatty acids, which is catalyzed by lipases and phospholipases (77). The accumulation of FFAs could be related to the activities of lipase and phospholipase in fish muscle, digestive organs and microorganisms, which were enhanced with storage time. The LVEO presence could produce lower FFAs formation due to the modification of the lipase environment leading to a partial inhibition of its catalytic action (31). Consistent with this research, the employment of LVEO inhibited FFAs formation in the refrigerated large yellow croaker samples.

### Water Distribution by LF NMR Analysis

LF-NMR is an effective way to evaluate the freshness of fish and MRI is also an assistive method to understand water migration in fish during storage (78). In this study the transverse relaxation time T_2_ showed a multi-exponential behavior, which suggests that the water is divided into populations in the muscle tissue. T_21_ ranged from 11.2 to 17.5 ms represent the fraction of strongly bound water. T_22_ (generally 100–400 ms) relates to the water within the organized protein structures (intra-myofibrillar) and T_23_ is the water in the space between myofibrils (extra-myofibrillar), which can be more easily mobilized by dripping or cooking and therefore susceptible to fish spoilage (79). The pT_21_, pT_22_, and pT_23_ correspond to the relative amount of bound water, immobilized water and free water, respectively (Table 1). Variations in relaxation times over time were expected in view of the changes in protein structure during deterioration. The pT_21_ rarely changed for large yellow croaker samples during refrigerated storage as the bound water held within highly organized myofibril structures (36). Some changes in pT_2_ suggested protein degradation in muscle tissue for large yellow croaker samples during refrigerated storage. The pT_22_ decreased progressively while pT_23_ increased for large yellow croaker samples during refrigerated storage. The CK had significant (*p* < 0.05) lower immobilized water content (from 95.35% at the beginning to 88.27% on 18th day) than that of other samples. However, no significant differences (*p* > 0.05) were shown in the immobilized water contents between LYC-0.30%LVEO and LYC-0.60%LVEO samples. The pT_23_ increased during refrigerated storage, however, the LVEO treated large yellow croaker samples had lower free water content than that of CK.

**Table 1:** Changes in water distribution in different treated large yellow croaker samples on 0 day, 9th day, and 18th day during refrigerated storage.

	**Time**	**CK**	**LYC-0.15%LVEO**	**LYC-0.30%LVEO**	**LYC-0.60%LVEO**
pT_21_/%	0 day	2.3 ± 0.1	2.3 ± 0.1	2.3 ± 0.1	2.3 ± 0.1
	9th day	2.2 ± 0.1a	2.2 ± 0.03a	2.1 ± 0.1a	2.2 ± 0.2a
	18th day	2.2 ± 0.04a	2.2 ± 0.1a	2.1 ± 0.1a	2.2 ± 0.1a
pT_22_/%	0 day	95.4 ± 0.4	95.4 ± 0.4	95.4 ± 0.4	95.4 ± 0.4
	9th day	93.3 ± 0.2a	93.4 ± 0.2a	94.0 ± 0.1b	94.0 ± 0.2b
	18th day	88.3 ± 0.3a	89.3 ± 0.3b	90.3 ± 0.3c	90.2 ± 0.2c
pT_23_/%	0 day	2.4 ± 0.3	2.4 ± 0.3	2.4 ± 0.3	2.4 ± 0.3
	9th day	4.4 ± 0.2a	4.4 ± 0.2a	3.9 ± 0.2b	3.8 ± 0.4b
	18th day	9.6 ± 0.3a	8.5 ± 0.2b	7.6 ± 0.4c	7.7 ± 0.2c

*Different letters in same day from different groups indicate a significant difference (p < 0.05)*.

**Figure 3:**
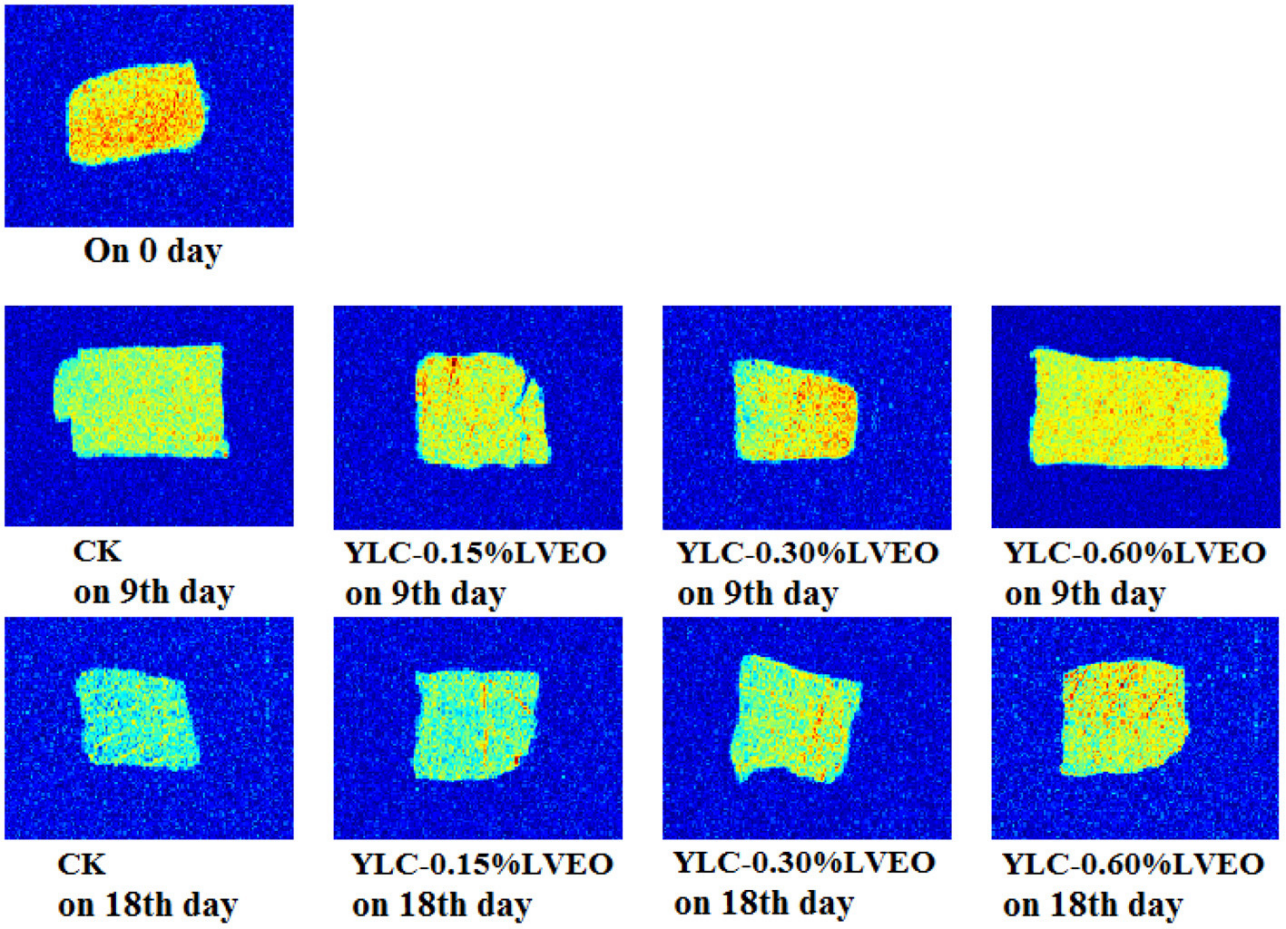
Results of magnetic resonance image (MRI) of large yellow croaker samples under different treatment during refrigerated storage.

MRI has attracted more and more attention for providing the visual information of spatial, internal morphological organization and molecular distribution in food matrix (73). Changes of water distribution of refrigerated large yellow croaker samples were investigated by T_1_ and T_2_ weighted images with MRI. Corresponding pseudo-color images are shown in Figure 3, in which red is the region with high proton signal density, and blue is the region with low proton signal density. There was no significant difference in MRI brightness of LVEO treated large yellow croaker samples on 9th day (Figure 3). Besides, the brightness of T_1_ and T_2_ images varied obscure and the brightness of the samples became darker and bluer during refrigerated storage. The color of CK samples was darker and bluer than other samples on 18th day and the brightness of LVEO treated samples were lighter compared with CK, which indicated the microstructure degradation and destruction of myofibril was more serious in CK sample (80).

### Changes in Hardness

**Figure 4:**
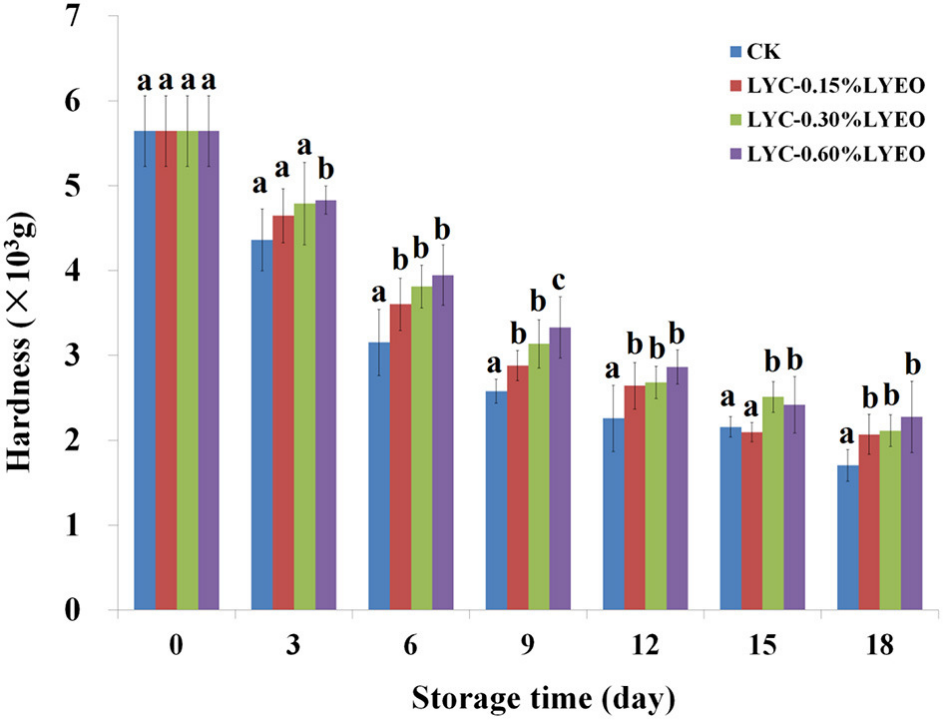
Changes in hardness of large yellow croaker samples during refrigerated storage. Different letters in same day from different groups indicate a significant difference (*p* < 0.05).

The hardness was 5.64 × 10^3^ g on 0 day (Figure 4) and decreased significantly (*p* < 0.05) in all large yellow croaker samples because the muscle became softer probably due to the autolytic activity of enzymes, the hydrolysis of protein and the destruction of connective tissue (40). The CK samples showed the fastest softening rate, losing about 69.86% of its hardness at the end, and LVEO treated samples had higher values of hardness than CK, which suggested LBG-SA active coatings incorporated with LVEO could decrease the loss of large yellow croaker samples hardness during refrigerated storage. Decreases in hardness of large yellow croaker during refrigerated storage was related to the enzymatic degradation of muscle proteins and thereafter accelerated by microbial activity (53). In the current study, LVEO treated samples reduced the loss of hardness by inhibiting the microbial growth with LVEO.

### Organoleptic Evaluation Results

**Figure 5:**
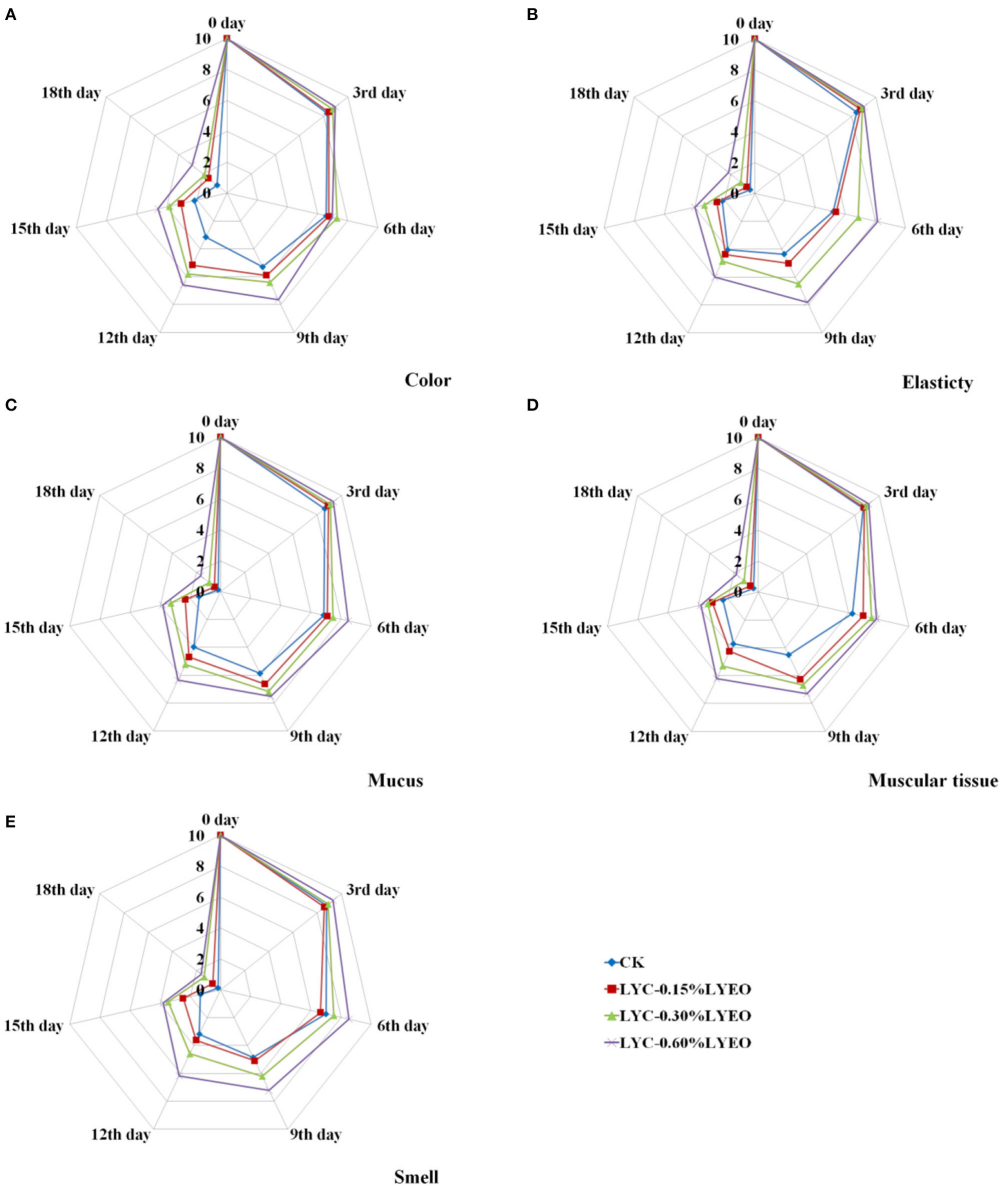
Changes in color **(A)**, elasticty **(B)**, mucus **(C)**, muscular tissue **(D)**, and smell **(E)** of large yellow croaker samples during refrigerated storage.

The organoleptic evaluation results including color, elasticty, mucus, muscular tissue and smell of large yellow croaker samples during refrigerated storage at 4°C are shown in Figure 5. At the beginning, all samples had high scores proving the good quality and the scores decreased significantly (*p* < 0.05) with the prolonging of storage time. However, the organoleptic results showed that the scores of the LVEO treated samples were significantly higher than that of CK. Therefore, the method of treating with the LBG-SA coating incorporated with LVEO could effectively delay the quality deterioration and maintain the organoleptic quality of large yellow croaker. At the end of storage, the scores of all the samples were lower than the limit value of 4 and they were considered as unacceptable for large yellow croaker samples in this research. The organoleptic results could directly show whether the large yellow croaker samples have gone spoiled during refrigerated storage. However, it took at least 2 or 3 days to get the chemical and microbiological results. The shelf life of perishable foods can be extended by reducing lipid oxidation and microbial reproduction. The inhibition of lipid oxidation can be attributed to the antioxidant properties of the active coatings, thereby reducing the production of unpleasant odors and flavors. The positive effects of EOs on the organoleptic properties of food have been demonstrated in some research (81). It should be noted that the smell of LVEO was also detected in the organoleptic evaluation; however, the influence on large yellow croaker was limited at this concentration. Besides, some of the observed changed could be due to inhomogeneities since the coating homogeneous and thickness was not measured.

## Conclusions

The LBG-SA active coatings incorporated with different LVEO concentrations were applied to evaluate the effects on quality improvement of large yellow croaker samples spoilage during refrigerated storage at 4°C for 18 days. This research focused on exploring the effect of LVEO on the quality of large yellow croaker samples during refrigerated storage and the large yellow croaker without LBG-SA active coating was not considered in the experiment. The results of microbiological and physicochemical analyses showed that the LBG-SA films incorporated with 0.30% LVEO and 0.60% LVEO emulsions treated large yellow croaker samples maintained better quality during refrigerated storage, which mainly due to that LVEO could effectively inhibit the growth of SSOs and resist to oxidation to extend the shelf life. LYC-0.30%LVEO and LYC-0.60%LVEO had similar effects in slowing down the spoilage of large yellow croaker; however, 0.60% LVEO addition gave the active coating solution a strong flavor. Therefore, 0.30% LVEO addition combined with refrigerated storage at 4°C could be suitable for maintaining the freshness of large yellow croaker samples and extended the shelf life.

## Data Availability Statement

The original contributions presented in the study are included in the article/Supplementary Material, further inquiries can be directed to the corresponding author/s.

## Author Contributions

BL, JM, and JX: conceptualization. BL, XW, XG, LZ, and XM: data curation. BL, XW, and JM: formal analysis. JX: funding acquisition and validation. BL, LZ, and JM: investigation. BL, LZ, JM, and JX: methodology. JM and JX: project administration, writing-review, and editing. BL and JM: software and writing-original draft. All authors contributed to the article and approved the submitted version.

## Conflict of Interest

LZ was employed by the company Shanghai Guo Qi Testing Services Technology Co., Ltd. The remaining authors declare that the research was conducted in the absence of any commercial or financial relationships that could be construed as a potential conflict of interest.
