# Supplementary material for: Shelf-Life Extension of Large Yellow Croaker (Larimichthys crocea) Using Active Coatings Containing Lemon Verbena (Lippa citriodora Kunth.) Essential Oil
Source: Front Nutr. 2021 Jul 20;8:678643. doi: 10.3389/fnut.2021.678643 (PMC8329554; doi:10.3389/fnut.2021.678643)
Supplement: Supplementary file 1 [file Table_1.DOCX]

Table 1. The analysis of chemical composition of Lemon verbena leaf essential oil by GC/MS.

| Peak No. | Retention time (min) | Chemical compound | Peak area (%) | Retention index |
| --- | --- | --- | --- | --- |
| 1 | 4.251 | β-Myrcene | 2.44 | 937 |
| 2 | 4.821 | d-Limonene | 10.36 | 1032 |
| 3 | 6.605 | Citronellal | 2.05 | 1153 |
| 4 | 6.729 | α-Isoneral | 2.59 | 1223 |
| 5 | 7.002 | β-Isoneral | 5.00 | 1226 |
| 6 | 7.869 | Neral | 23.75 | 1242 |
| 7 | 8.018 | Geraniol | 22.01 | 1251 |
| 8 | 8.290 | Citral | 31.79 | 1271 |
